# Cross-Sectional Comparative Assessment of Periodontal Status in Diabetic and Non-Diabetic Individuals Within a Romanian Cohort

**DOI:** 10.3390/jcm14228199

**Published:** 2025-11-19

**Authors:** Iulia Alexa, Ramona Dumitrescu, Vlad Tiberiu Alexa, Simona Popescu, Vanessa Bolchis, Lucian Floare, Octavia Balean, Atena Galuscan, Daniela Jumanca

**Affiliations:** 1 Translational and Experimental Clinical Research Centre in Oral Health, Clinic of Preventive, Community Dentistry and Oral Health, University of Medicine and Pharmacy “Victor Babes”, 300040 Timisoara, Romania; iulia.alexa@umft.ro (I.A.); dumitrescu.ramona@umft.ro (R.D.); vanessa.bolchis@umft.ro (V.B.); lucian.floare@umft.ro (L.F.); balean.octavia@umft.ro (O.B.); galuscan.atena@umft.ro (A.G.); jumanca.daniela@umft.ro (D.J.); 2 Department of Dentistry, Faculty of Dental Medicine, “Vasile Goldis” Western University of Arad, 310045 Arad, Romania; 3 Clinic of Preventive, Community Dentistry and Oral Health, Department I, University of Medicine and Pharmacy “Victor Babes”, Eftimie Murgu Sq. No 2, 300041 Timisoara, Romania; 4 Second Department of Internal Medicine, University of Medicine and Pharmacy “Victor Babes”, 300041 Timisoara, Romania; 5 Department of Diabetes, “Pius Brinzeu” Emergency Hospital, 300723 Timisoara, Romania

**Keywords:** diabetes mellitus, periodontitis, periodontal index, dental plaque, inflammation, glycated hemoglobin A, smoking, Romania

## Abstract

**Background/Objectives:** Periodontitis and diabetes mellitus are known to exert a bidirectional pathogenic influence characterized by metabolic dysregulation, exaggerated inflammatory response, and accelerated periodontal breakdown. Despite the increasing prevalence of both conditions in Eastern Europe, comparative clinical data from Romanian populations remain limited. This study aimed to assess differences in periodontal status, systemic comorbidities, smoking exposure, and local periodontal risk factors between diabetic and non-diabetic individuals in a Romanian cohort. **Methods:** This cross-sectional study included 345 adults (152 with diabetes; 193 non-diabetic individuals) all diagnosed with periodontitis. Although participants were recruited to achieve comparable distributions of sex and smoking status, individuals with diabetes were significantly older than controls. Full-mouth periodontal examination was performed following the 2018 EFP/AAP classification criteria. Clinical parameters included probing pocket depth (PPD), clinical attachment level (CAL), plaque index (PI), and bleeding on probing (BOP). Periodontal staging and grading were determined, and local risk factors were recorded using a multiple-response approach. Systemic comorbidities and smoking exposure were also evaluated. Statistical analyses involved *t*-tests, chi-squared tests, and Spearman correlations; significance was set at *p* < 0.05. **Results:** Diabetic patients exhibited significantly higher mean PPD (4.33 ± 1.22 mm vs. 3.70 ± 0.86 mm; mean difference 0.63 mm, 95% CI: 0.45–0.81), greater CAL (mean difference 0.19 mm, 95% CI: 0.01–0.37), and elevated PI and BOP (PI mean difference 18.97%, 95% CI: 12.44–25.50; BOP mean difference 17.23%, 95% CI: 10.86–23.60). Advanced stages (III–IV) and Grade C periodontitis were more prevalent among diabetic individuals. Diabetic participants also presented a higher burden of local plaque-retentive factors and systemic comorbidities (notably hypertension and cardiovascular disease), as well as higher smoking intensity. **Conclusions:** Diabetes mellitus is associated with markedly poorer periodontal status, greater systemic disease burden, and increased prevalence of plaque-retentive local factors. These findings reinforce the bidirectional relationship between diabetes and periodontitis and support the integration of periodontal assessment into metabolic disease management protocols.

## 1. Introduction

Periodontal disease represents one of the most prevalent chronic inflammatory conditions affecting humans and is increasingly recognized as a complex, multifactorial disorder rather than a simple bacterial infection. It results from a dynamic interaction between pathogenic subgingival biofilms and the host’s immune–inflammatory response, influenced by genetic, behavioral, and environmental factors [1]. Historically, the relationship between oral and systemic health has been debated since the early 20th century, evolving from the “focal infection” theory to contemporary evidence-based research linking chronic periodontitis to several systemic diseases [1]. The pathogenesis of periodontitis involves dysbiotic bacterial communities—such as Porphyromonas gingivalis, Tannerella forsythia, and Treponema denticola—that stimulate an exaggerated host immune response, leading to tissue destruction mediated by pro-inflammatory cytokines, eicosanoids, and matrix metalloproteinases [2].

Beyond being an oral infection, periodontal disease is now understood as a chronic inflammatory condition with systemic repercussions. Contemporary evidence shows that the relationship between oral and systemic health is biologically grounded rather than coincidental, with inflammatory pathways and microbial dissemination serving as shared mechanisms linking periodontal and systemic diseases such as diabetes, cardiovascular disease, and rheumatoid arthritis [3].

Beyond microbial challenge, systemic conditions such as diabetes mellitus and lifestyle factors like smoking play pivotal roles as disease modifiers, amplifying inflammatory pathways and accelerating periodontal tissue breakdown. Consequently, periodontitis has emerged not only as a major cause of tooth loss but also as a significant public health concern due to its global prevalence and its close association with systemic disorders, particularly diabetes mellitus [1].

It is well established that periodontal inflammation is markedly more severe in individuals with chronic hyperglycemia—including those with prediabetes or poorly controlled type 2 diabetes mellitus (T2DM)—compared with normoglycemic individuals [4]. One mechanistic explanation for this relationship is that sustained hyperglycemia upregulates the expression of toll-like receptors (TLRs) and enhances the production of pro-inflammatory cytokines, such as interleukin-1β (IL-1β) and tumor necrosis factor-α (TNF-α), within periodontal tissues, thereby amplifying the local inflammatory response [5]. Furthermore, chronic hyperglycemia contributes to oxidative stress, disturbing the balance between the generation and neutralization of reactive oxygen species (ROS). In parallel, prolonged exposure to high glucose levels promotes the formation and accumulation of advanced glycation end-products (AGEs) in periodontal structures, which further aggravate inflammation and tissue destruction through receptor-mediated pathways [6]. Recent reviews emphasize that this bidirectional link between diabetes and periodontitis is supported by multiple biological mechanisms, including altered neutrophil function, AGE accumulation, endothelial dysfunction, and low-grade systemic inflammation that exacerbates insulin resistance [3]. Notably, effective periodontal therapy has been associated with mean HbA1c reductions of approximately 0.4–0.6%, underscoring the clinical relevance of periodontal control in diabetes management [3].

The primary goal of diabetes management is to minimize the risk of microvascular and macrovascular complications through sustained glycemic control, typically reflected by glycated hemoglobin (HbA1c) levels below individualized targets. In general, HbA1c values under 53 mmol/mol (7.0%) indicate good metabolic control, while non-diabetic individuals usually exhibit values around 37 mmol/mol (5.5%). Each 1% reduction in HbA1c (approximately 11 mmol/mol) is associated with substantial decreases in diabetes-related mortality (21%), myocardial infarction (14%), and microvascular complications (37%). Treatment strategies for diabetes require lifelong management, including dietary regulation, weight control, physical activity, pharmacologic therapy (oral hypoglycemics and/or insulin), and adherence to individualized care plans. Achieving and maintaining optimal glycemic control is therefore crucial not only for systemic health but also for mitigating oral inflammatory burden in diabetic patients [7].

Clinical evidence also indicates that effective glycemic control—achieved through dietary management and/or antihyperglycemic therapy—significantly reduces the severity of periodontal disease. Improvements have been consistently observed across both clinical parameters (including plaque index [PI], bleeding on probing [BOP], probing depth [PD], and clinical attachment loss [CAL]) and radiographic indicators such as marginal bone loss (MBL), in patients with prediabetes and type 2 diabetes mellitus (T2DM) [6,8]. Conversely, periodontitis itself may adversely affect glycemic control, while conventional periodontal therapy has been shown to improve metabolic regulation and reduce the risk of diabetic comorbidities. These findings underscore the bidirectional relationship between diabetes and periodontal disease, whereby each condition can exacerbate the other [9].

Extensive evidence from international consensus reports and systematic reviews supports this bidirectional association [10,11,12,13]. Diabetes, particularly when poorly controlled, increases both the risk and severity of periodontitis through mechanisms involving hyperglycemia-induced systemic inflammation, oxidative stress, impaired neutrophil function, and the accumulation of advanced glycation end-products that hinder tissue repair. Conversely, periodontitis contributes to worsened glycemic control by promoting chronic systemic inflammation and transient bacteremia, leading to insulin resistance [13].

Findings from the 2017 EFP–IDF Workshop on Periodontitis and Diabetes and the more recent WONCA Europe–EFP 2022 Workshop confirmed that this association is consistent, independent, and clinically significant. Evidence synthesized from multiple longitudinal and intervention studies demonstrated that non-surgical periodontal therapy can lead to meaningful reductions in HbA1c levels, ranging between 0.3% and 0.6% within 3–12 months, comparable to adding a second oral hypoglycemic drug to standard therapy [14,15,16]. These findings reinforce the view that periodontal health plays an integral role in systemic disease management and should be recognized as a “sixth complication of diabetes”.

Despite growing international recognition of this interconnection, integrated care remains limited in routine clinical practice. The separation of dentistry from general medicine has historically hindered early detection of diabetes in dental settings and vice versa. Recent international perspectives advocate transdisciplinary collaboration between medical and dental professionals to improve early diagnosis, shared management, and patient outcomes [17].

Despite this, the regional context remains underexplored, particularly in Eastern Europe, where the prevalence of diabetes continues to rise. From a public health perspective, the high and steadily growing prevalence of diabetes in Romania makes this interrelationship particularly relevant. The PREDATORR national epidemiological study, conducted on 2728 adults aged 20–79 years, reported an age- and sex-adjusted prevalence of 11.6% for diabetes and 16.5% for prediabetes, indicating that more than one in four Romanian adults exhibit impaired glucose regulation [18]. These data emphasize the growing burden of metabolic disorders and highlight the need to better understand their oral health implications in this population.

Recent large-scale epidemiologic data have further quantified the association between periodontal disease and systemic conditions such as diabetes and hypertension, confirming their moderate but clinically relevant co-occurrence in general populations [19]. In parallel, contemporary primary-care research emphasizes the need for integrated medical–dental strategies, including validated oral-health screening tools for diabetes care providers, to facilitate early detection and referral [20]. Collectively, these findings reinforce the shift toward interdisciplinary approaches that address oral and systemic inflammation within shared care frameworks.

Building upon these international trends, recent national and regional studies have also highlighted the complex relationship between glycemic control, systemic inflammation, and periodontal health. Data from Romanian cohorts have contributed to this field by showing that poor periodontal status negatively affects quality of life in patients with diabetes and is associated with elevated inflammatory markers such as IL-1β and TNF-α [21,22]. Behavioral and lifestyle factors, including oral hygiene practices and smoking, have also been shown to exacerbate this relationship [23].

However, most of these studies focus primarily on diabetic populations, without directly comparing them to non-diabetic individuals using standardized full-mouth periodontal evaluation protocols. Comparative analyses that simultaneously account for periodontal status, systemic comorbidities, and behavioral risk factors in Romanian adults are still limited.

In this context, the present study aims to address this gap by comparing periodontal outcomes between diabetic and non-diabetic individuals within a Romanian cohort, using current EFP/AAP classification criteria. By aligning local data with international findings, this study seeks to provide a more comprehensive understanding of the diabetes–periodontitis relationship in an Eastern European setting and to support the development of more integrated preventive and therapeutic strategies.

## 2. Materials and Methods

### 2.1. Study Design and Ethical Considerations

This cross-sectional, observational, and correlational study was conducted between May and December 2024 at the Department of Oral Health in Timisoara, Faculty of Dental Medicine, “Victor Babeș” University of Medicine and Pharmacy, Timișoara, Romania. The study protocol was reviewed and approved by the Ethics Committee of the “Victor Babeș” University of Medicine and Pharmacy, Timișoara (approval no. 05/30 January 2024), and all participants provided written informed consent before inclusion. The study adhered to the principles of the Declaration of Helsinki.

### 2.2. Study Population

The study included patients with and without diabetes mellitus attending the Department of Oral Health, Faculty of Dental Medicine, Timișoara. Data were obtained from two distinct yet methodologically comparable recruitment sources:

- patients with a confirmed diagnosis of diabetes mellitus (predominantly type 2, with a smaller number of type 1 cases) referred from the Outpatient Diabetes Care Facility (Pius Brînzeu County Emergency Clinical Hospital, Timișoara, Romania) for periodontal evaluation; and
- systemically healthy individuals (non-diabetic individuals) presenting to the Department of Oral Health for routine dental examination.

No a priori sample size calculation was performed, as the study followed a convenience sampling approach and included all eligible patients during the recruitment period. Therefore, the analyses should be interpreted as exploratory.

All participants had a clinical diagnosis of periodontitis at the time of examination. Therefore, the non-diabetic group should be interpreted as a comparison group of non-diabetic periodontal patients rather than periodontally healthy controls.

Although participants were selected to achieve comparable distributions in terms of sex and smoking status, individuals with diabetes were significantly older than controls. This difference is reported transparently in the Results and acknowledged as a potential source of selection bias. However, both groups underwent the same clinical protocol and diagnostic criteria to ensure methodological consistency. Differences in age and smoking intensity between groups were considered when interpreting the findings and are discussed as potential confounding factors in Section 4.

### 2.3. Inclusion and Exclusion Criteria

Participants eligible for inclusion were adults aged 18 years or older who were able and willing to provide written informed consent and who presented for periodontal evaluation at the study centers. For the diabetic cohort, a confirmed diagnosis of type 2 diabetes mellitus was required, while all participants were required to have clinical evidence of periodontal disease established through a comprehensive oral examination.

Patients were excluded if they were pregnant or lactating, had psychological or cognitive impairments that could compromise their ability to provide informed consent, or had received systemic antibiotic or anti-inflammatory therapy within the previous three months, as such treatments could influence inflammatory and periodontal parameters. Additional exclusion criteria included the absence of periodontal disease upon clinical examination or the refusal to participate in the study.

After applying the inclusion and exclusion criteria, diabetic and non-diabetic participants were recruited using a frequency-matching approach to achieve comparable distributions by sex and smoking status. However, age differences between groups persisted and were reported transparently.

### 2.4. Examiner Training and Calibration

Prior to data collection, all resident examiners underwent a structured calibration program supervised by a reference examiner. The program included (i) a refresher session on the full-mouth protocol and recording rules per the 2018 EFP/AAP criteria; (ii) hands-on practice on volunteer patients followed by feedback; and (iii) duplicate measurements on a set of patients not included in the main analysis. For reliability assessment, each examiner recorded PPD and CAL at six sites/tooth using a CPITN probe (Aesculap AG, Tuttlingen, Germany) with 0.5 mm markings. Duplicate measurements were repeated after ≥48 h to assess intra-examiner reliability; a second examiner performed independent readings on the same sites to assess inter-examiner reliability. Reliability was summarized with intraclass correlation coefficients (ICC, two-way random effects, absolute agreement) for continuous outcomes. Pre-specified acceptance thresholds were ICC ≥ 0.80 for both PPD and CAL. When discrepancies > 1 mm were observed, targeted retraining was performed before enrollment commenced.

### 2.5. Data Collection and Clinical Examination

All participants completed a standardized questionnaire designed to gather information on demographic characteristics, including age, sex, and place of residence, as well as medical history and smoking habits.

Following completion of the questionnaire, each subject underwent a comprehensive full-mouth periodontal examination performed by calibrated resident dentists under the supervision of experienced faculty members.

The clinical assessment followed standardized diagnostic protocols and was carried out using a Community Periodontal Index of Treatment Needs (CPITN) probe, equipped with calibrated markings at 3.5, 5.5, 8.5, and 11.5 mm to ensure accurate measurement of periodontal pockets. A CPITN periodontal probe (Aesculap AG, Tuttlingen, Germany) (0.5 mm markings) was used strictly as the measurement instrument for probing pocket depth and clinical attachment level at six sites per tooth; the CPITN/CPI index was not applied in this study. Periodontal parameters were assessed at six sites per tooth and included probing pocket depth (PPD), clinical attachment level (CAL), plaque index (PI), and bleeding on probing (BOP). Each case was further classified according to the 2018 periodontal disease classification system proposed by Caton et al. (2018) [24] which specifies disease staging and grading. All measurements were recorded digitally using the Berne Periodontal Chart platform (Berner Fachhochschule, Bern, Switzerland) to ensure consistency, traceability, and standardized data entry across examiners.

Local plaque-retentive and periodontal risk factors were assessed clinically and categorized into ten predefined classes: (1) visible dental plaque, (2) supra- or subgingival calculus, (3) cervical or root caries, (4) faulty or overhanging restorations, (5) maladapted fixed or removable prostheses, (6) endo-periodontal lesions, (7) edentulous spaces, (8) malpositioned or rotated teeth, (9) signs of traumatic occlusal forces (e.g., fremitus, tooth mobility inconsistent with attachment loss, wear facets), and (10) peri-implant inflammation or bone loss where applicable. Each factor was recorded dichotomously (present/absent) per tooth.

Examiners were trained to identify these conditions during the calibration session used for periodontal measurements, using standardized visual-tactile criteria and clinical photographs. Because multiple local factors may coexist and concurrently contribute to periodontal breakdown in the same patient, a multiple-response approach was applied: for each participant, all present factors were recorded, and the cumulative number of local risk factors per individual was calculated.

### 2.6. Statistical Analysis

Data analysis was performed using IBM SPSS Statistics, Version 23 (IBM Corp., Armonk, NY, USA). The study was conceived as an exploratory and descriptive cross-sectional analysis; therefore, statistical tests were used to assess crude associations rather than to establish causality. Continuous variables (including percentage indices such as PI and BOP) were first tested for normality using the Shapiro–Wilk test. Normally distributed variables were expressed as mean ± standard deviation (SD), while non-normally distributed data were presented as median and interquartile range (IQR). IQR was defined as the range between the 25th and 75th percentiles (Q1–Q3), representing the central dispersion of values. Categorical variables were expressed as frequencies and percentages. PPD and CAL followed approximately normal distributions and were analyzed using parametric tests (independent-samples *t*-test), whereas PI and BOP showed non-normal distributions and were compared using the non-parametric Mann–Whitney U test.

Independent-samples *t*-tests or Mann–Whitney U tests were applied to compare periodontal indices (PPD, CAL, PI, BOP) between diabetic and non-diabetic groups, depending on data distribution. Chi-squared tests were used to evaluate categorical variables such as periodontal stage and grade. Spearman’s rank correlations assessed associations between glycemic control (HbA1c levels, where available) and periodontal parameters. Effect sizes for between-group comparisons were calculated and reported as mean or median differences with corresponding 95% confidence intervals, providing a quantitative measure of the magnitude and precision of observed associations beyond *p*-values.

No multivariable regression models were applied, and therefore, potential confounders such as age and smoking were not adjusted beyond stratification and transparent reporting in baseline characteristics. The influence of smoking intensity was examined descriptively, but no adjustment was applied for its potential confounding effect on periodontal outcomes. A two-tailed *p*-value < 0.05 was considered statistically significant.

### 2.7. Use of GenAI in Writing

AI assistance (ChatGPT version 5.1, OpenAI) was used exclusively to improve language clarity, grammar, and readability of the manuscript. All scientific content, study design, analyses, and interpretations are entirely the authors’ own work.

## 3. Results

### 3.1. Study Population

A total of 345 participants were included, comprising 152 individuals with diabetes and 193 non-diabetic individuals. The analysis showed that age differed significantly between the two groups (*p* < 0.001), with individuals in the diabetic cohort being older than those in the non-diabetic cohort (mean age: 58.3 ± 9.7 years vs. 49.6 ± 10.2 years, respectively). In contrast, the proportion of males and females and the distribution of urban versus rural residence were comparable between the groups (*p* > 0.05), indicating demographic balance in these parameters. Baseline demographic and clinical characteristics of diabetic and non-diabetic periodontal patients are presented in Table 1, including complete numerical summaries (mean ± SD or median [IQR] for continuous variables and n [%] for categorical data) to allow full verification of group comparability. HbA1c values were available for 128 diabetic participants (84.2%) and were retrieved from medical records within three months prior to the periodontal examination. Baseline demographic and clinical characteristics of diabetic and non-diabetic periodontal patients are presented in Table 1, including complete numerical summaries (mean ± SD or median [IQR] for continuous variables and n [%] for categorical data) to allow full verification of group comparability. HbA1c values were available for 128 diabetic participants (84.2%) and were retrieved from medical records within three months prior to the periodontal examination.

### 3.2. Systemic Comorbidities

Among the diabetic cohort (n = 152), 138 patients (90.8%) were diagnosed with type 2 diabetes mellitus (T2DM), while 14 patients (9.2%) had type 1 diabetes mellitus (T1DM). Systemic comorbidities were markedly more prevalent in the diabetic group compared with non-diabetic participants. Hypertension was the most common systemic condition, affecting 102 diabetic patients (67.1%) versus 38 non-diabetic participants (19.7%). Cardiovascular diseases (excluding hypertension) were present in 28 diabetic individuals (18.4%) and 11 non-diabetic individuals (5.7%), while respiratory conditions such as asthma were recorded in 9 diabetic patients (5.9%) and 4 non-diabetic patients (2.1%). Other systemic disorders (including renal, hepatic, rheumatologic, or endocrine diseases) were identified in 21 diabetic participants (13.8%) and 10 non-diabetic participants (5.2%). In contrast, the absence of systemic disease was documented in 145 non-diabetic individuals (75.1%), compared with only 8 diabetic patients (5.3%), highlighting a significantly higher multimorbidity burden in individuals with diabetes (*p* < 0.001).

### 3.3. Periodontal Clinical Parameters

Patients with diabetes exhibited significantly poorer periodontal health compared with non-diabetic individuals. The mean probing pocket depth (PPD) was 4.33 ± 1.22 mm in the diabetic group versus 3.70 ± 0.86 mm in the non-diabetic group (mean difference: 0.63 mm, 95% CI: 0.45–0.81; *p* < 0.001). The mean clinical attachment level (CAL) was 1.89 ± 4.70 mm in diabetics compared with 1.70 ± 4.29 mm in non-diabetic individuals (mean difference: 0.19 mm, 95% CI: 0.01–0.37; *p* = 0.043). Inflammatory indices were also markedly elevated among patients with diabetes, with a mean plaque index of 44.01% ± 29.46 compared to 25.04% ± 26.43 (mean difference: 18.97%, 95% CI: 12.44–25.50; *p* < 0.001), and bleeding on probing (BOP) was 43.84% ± 28.76 vs. 26.61% ± 27.44 (mean difference: 17.23%, 95% CI: 10.86–23.60; *p* < 0.001). These findings indicate a significantly higher inflammatory burden and greater periodontal breakdown in the diabetic cohort. Full descriptive statistics are presented in Table 2. CAL values showed considerable variability within each group, reflecting the coexistence of mild and advanced attachment loss among participants, which contributed to the wide dispersion observed in CAL measurements.

**Figure 1:**
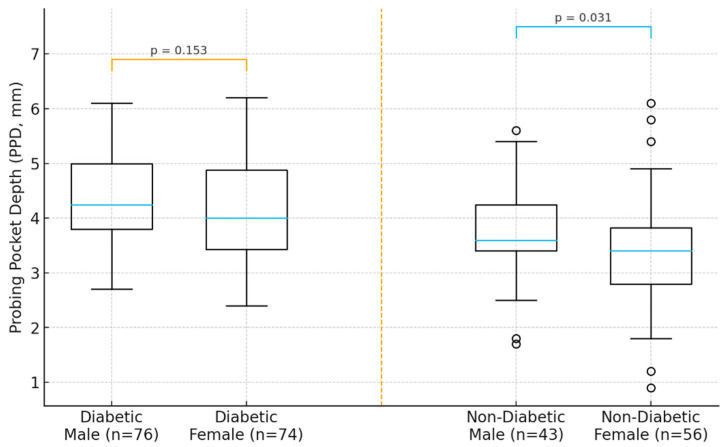
Distribution of periodontal parameters (PPD and CAL) by sex in diabetic and non-diabetic participants.

In the diabetic cohort, male participants exhibited higher mean PPD and CAL compared with females, suggesting a more severe periodontal profile in men. A similar but less pronounced pattern was observed in the non-diabetic group. Age demonstrated a positive trend with periodontal severity, with older individuals showing deeper pockets and greater attachment loss in both cohorts. These descriptive trends indicate that sex may act as a modifying factor in the periodontal response, potentially interacting with systemic metabolic status (Figure 1).

### 3.4. Periodontitis Staging and Grading

The distribution of periodontal stages and grades according to the 2018 EFP/AAP classification is presented in Table 3. Diabetic patients more frequently exhibited advanced periodontitis, with Stage III–IV recorded in 59.9% of diabetics versus 48.1% of non-diabetic periodontal individuals (χ^2^ = 10.56, *p* = 0.001). Conversely, Stage II disease was more common among non-diabetic individuals. Similarly, Grade C (rapid progression) was markedly more prevalent among diabetic participants (78.7% vs. 63.7%; χ^2^ = 8.29, *p* = 0.004), whereas Grade B predominated in the non-diabetic group. These findings confirm a higher severity and faster progression of periodontal destruction in patients with diabetes.

The distribution of periodontal staging demonstrated a significantly higher prevalence of Stage IV periodontitis in the diabetic group compared with non-diabetic controls (59.7% vs. 48.1%, χ^2^ = 16.03, *p* = 0.001). Similarly, Grade C, indicative of rapid progression, was markedly more common among diabetic participants (78.7% vs. 63.7%, χ^2^ = 13.21, *p* = 0.004). Correlation analysis within the diabetic cohort revealed a statistically significant negative association between HbA1c and plaque index (Spearman’s r = −0.234, *p* = 0.034), while no significant correlations were found for probing pocket depth, clinical attachment loss, or bleeding on probing (*p* > 0.05).

### 3.5. Correlation Between Glycemic Control and Periodontal Parameters

**Figure 2:**
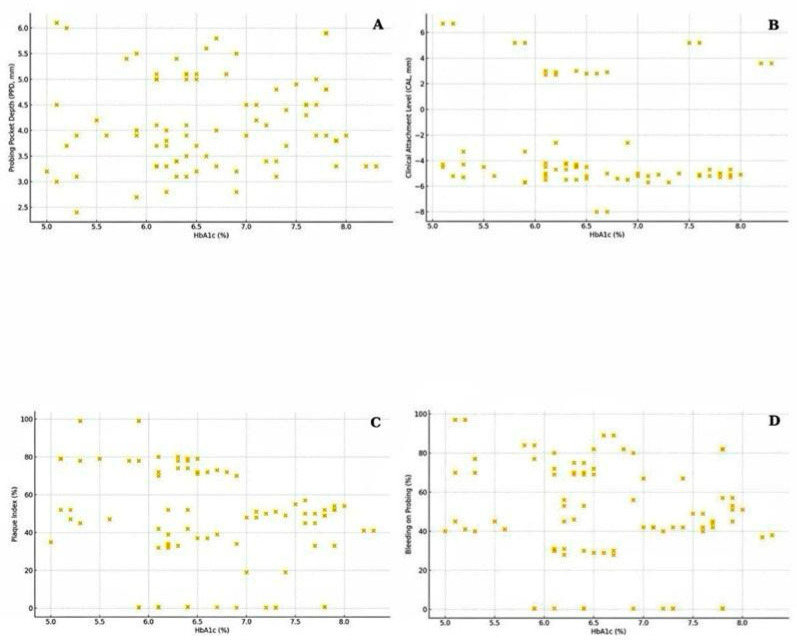
Scatterplots showing the association between glycated hemoglobin (HbA1c) and (**A**) probing pocket depth (PPD), (**B**) clinical attachment level (CAL), (**C**) plaque index (PI), and (**D**) bleeding on probing (BOP) in the diabetic group.

The relationship between glycemic control (HbA1c) and periodontal measures was further explored using Spearman correlation analysis and visualized in scatterplots (Figure 2). A statistically significant negative correlation was observed between HbA1c and plaque index (Spearman’s r = −0.234, *p* = 0.034), indicating that patients with poorer glycemic control tended to present with lower visible plaque accumulation, potentially reflecting altered oral hygiene behaviors or salivary changes associated with diabetes. However, this inverse association should be interpreted with caution, as it may reflect behavioral or monitoring factors rather than a biological effect. Patients with higher HbA1c levels often receive more frequent medical and dental supervision, including oral hygiene reinforcement, which could temporarily lower plaque accumulation scores. Although correlations between HbA1c and probing pocket depth (PPD), clinical attachment level (CAL), and bleeding on probing (BOP) did not reach statistical significance, a positive directional trend was noted, suggesting that increasing HbA1c may be associated with greater periodontal tissue breakdown and inflammatory burden. These findings align with current evidence supporting the role of hyperglycemia in the pathogenesis of periodontal disease.

### 3.6. Smoking Exposure

The intensity of tobacco use differed significantly between groups. The median number of cigarettes smoked per day was 15 (IQR: 10–20) in the diabetic group compared with 5 (IQR: 0–10) in the non-diabetic group (*p* < 0.001, Mann–Whitney U test) (Table 3). This indicates that individuals with diabetes were more likely to be current smokers and reported substantially higher daily tobacco exposure.

### 3.7. Medication Profile

In the diabetic cohort (n = 152), the majority of patients were undergoing pharmacological treatment for glycemic control. Metformin-based therapy (including Metformin, Siofor, and Glucophage) was prescribed to 108 patients (71.1%), while insulin therapy (either as monotherapy or combined with oral agents) was reported by 42 patients (27.6%). Combination therapy involving both insulin and oral antidiabetic agents was recorded in 31 individuals (20.4%), reflecting the predominance of advanced or long-standing diabetes.

Cardiovascular medications were also frequently used among diabetic participants: antihypertensive agents were prescribed to 97 patients (63.8%), lipid-lowering drugs (mainly statins) to 84 patients (55.3%), and antiplatelet or anticoagulant therapy (e.g., Aspenter, Plavix, Trombex) to 19 patients (12.5%), indicating widespread secondary cardiovascular prevention. By contrast, the non-diabetic group (n = 193) was largely medication-free, with 138 participants (71.5%) reporting no current systemic therapy. Among those under treatment, antihypertensive agents were the most common (29 patients, 15.0%), followed by antiplatelet agents (8 patients, 4.1%) and respiratory medications (5 patients, 2.6%). These findings illustrate the substantially greater systemic disease burden and pharmacological complexity among diabetic individuals, consistent with the clustering of cardiometabolic comorbidities in this population. Complete data are summarized in Table 4.

### 3.8. Local Periodontal Risk Factors

Local periodontal risk factors were frequently observed in both groups; however, their prevalence was substantially higher among individuals with diabetes (Table 5). The most common factor in the diabetic cohort was dental plaque accumulation, detected in 142 patients (93.4%), followed by subgingival calculus (128 patients, 84.2%) and faulty restorative or prosthetic work (97 patients, 63.8%). Cervical or root caries were present in 91 diabetic individuals (59.9%), and prosthetic maladaptation in 62 cases (40.8%). Less frequent but clinically relevant factors included malpositioned teeth (49 patients, 32.2%).

In the non-diabetic group, plaque remained the primary local factor (158 patients, 81.9%), followed by calculus (131 patients, 67.9%), faulty restorations (88 patients, 45.6%), and cervical caries (76 patients, 39.4%). The cumulative number of local risk factors per individual was significantly higher among diabetic participants (median 4 vs. 2 factors; *p* < 0.001), indicating a more complex ecological environment conducive to persistent inflammatory stimulation and accelerated periodontal breakdown. This increased load of plaque-retentive local factors in diabetic subjects aligns with their greater periodontal severity and higher staging and grading distribution observed in previous analyses.

## 4. Discussion

This study provides new clinical evidence on the periodontal status of diabetic and non-diabetic individuals within a Romanian population, contributing to the growing body of literature on the bidirectional relationship between metabolic dysregulation and periodontal disease. Our findings are consistent with the conclusions of the EFP–IDF 2017 Focused Workshop and the WONCA Europe–EFP 2022 consensus, which established a robust evidence base for this association [12,25]. Diabetic patients demonstrated significantly poorer periodontal parameters, consistent with pathophysiological mechanisms previously described in consensus reports, including hyperglycemia-induced oxidative stress, altered immune response, and delayed tissue repair [13]. Moreover, the distribution of periodontal staging and grading observed in our cohort suggests that metabolic dysregulation may be associated with more advanced periodontal disease. Individuals with diabetes were more likely to present with Stage III–IV and Grade C periodontitis, reflecting more advanced and rapidly progressing forms of the disease. The positive association between elevated HbA1c levels and periodontal severity parameters further supports the hypothesis that poor glycemic control contributes to periodontal breakdown through inflammatory and immune-mediated pathways. Collectively, these data are consistent with the concept of a bidirectional association between diabetes and periodontitis and underscore the importance of glycemic control as a key modifiable determinant of periodontal and systemic health.

Furthermore, the modest improvements in periodontal status observed among non-diabetic individuals suggest that glycemic control may act as a modifiable determinant of oral inflammation. These data are congruent with meta-analyses showing clinically meaningful HbA1c reductions (0.3–0.6%) following periodontal therapy [11,16]. reinforcing the importance of interdisciplinary care between medical and dental teams.

The substantially higher prevalence of systemic comorbidities among diabetic participants reflects the clustering of chronic non-communicable diseases characteristic of metabolic syndrome. Hypertension and cardiovascular disease were significantly overrepresented in the diabetic cohort, consistent with shared inflammatory and endothelial dysfunction pathways that contribute to both systemic and periodontal tissue damage. Previous studies have demonstrated that the coexistence of diabetes with cardiovascular comorbidities amplifies oxidative stress and promotes a pro-inflammatory state, thereby accelerating periodontal breakdown and impairing treatment response [26]. In addition, an unexpected inverse correlation between HbA1c and plaque index was identified in our cohort. This finding should be interpreted with caution, as it likely reflects behavioral and monitoring factors rather than a biological relationship. In this clinical context, the inverse association observed between HbA1c and plaque index should be interpreted with caution. Rather than indicating a protective effect, this trend may reflect intensified medical and dental supervision among patients with poorer glycemic control, who often receive more frequent periodontal maintenance procedures that temporarily reduce visible plaque without altering underlying inflammation. Furthermore, diabetes-associated alterations in salivary flow and composition may influence plaque adherence and clinical detection. These findings underscore the complexity of interpreting plaque as a standalone marker in diabetic populations and highlight the need to consider systemic disease burden, medication use, and oral care behaviors when evaluating periodontal status. Longitudinal studies are required to determine whether this inverse trend represents a clinically relevant phenomenon or a statistical variation driven by multifactorial influences within a metabolically compromised cohort.

In the present study, individuals with diabetes reported substantially higher levels of tobacco exposure compared with their non-diabetic counterparts, suggesting that smoking may act as an additional modifier exacerbating periodontal breakdown. Smoking is a well-established environmental risk factor known to impair vascularization, reduce neutrophil function, and increase systemic oxidative stress, mechanisms that synergistically amplify the inflammatory burden already imposed by chronic hyperglycemia. Moreover, nicotine and combustion products have been shown to promote advanced glycation end-product (AGE) formation and upregulate pro-inflammatory cytokines, further accelerating connective tissue degradation and alveolar bone loss in patients with diabetes [27]. The combined presence of diabetes and smoking has been associated with more severe forms of periodontitis, faster rates of attachment loss, and poorer therapeutic outcomes compared with either factor alone [28]. Therefore, the elevated smoking intensity observed in the diabetic cohort of this study likely contributed to the higher prevalence of Stage IV and Grade C periodontitis in this group, underscoring the need for smoking cessation to be integrated as a core component of periodontal and metabolic disease management. Because the present analysis was unadjusted, the independent effects of diabetes and smoking cannot be fully separated. Future studies employing multivariable or stratified designs are needed to clarify the extent to which smoking intensity versus metabolic control contributes to periodontal severity.

The medication profile observed in this study is consistent with current international guidelines for the management of diabetes and associated cardiovascular risk. The predominance of metformin and insulin use among diabetic individuals reflects the chronic nature and advanced metabolic control requirements of this cohort. The high rate of antihypertensive and statin therapy further emphasizes the systemic pro-inflammatory and pro-atherogenic milieu common in diabetes, which may exacerbate periodontal breakdown. Pharmacological agents such as antihypertensives and lipid-lowering drugs can modulate vascular responses and salivary flow, potentially affecting periodontal tissue repair. These findings support the hypothesis that periodontitis in diabetic patients is influenced not only by hyperglycemia but also by the broader cardiometabolic disease network, highlighting the need for integrated medical-dental management strategies.

From a clinical perspective, dental practitioners should routinely record a complete medical history for all patients and pay particular attention to glycemic control in individuals with diabetes. Inquiry about the patient’s usual antidiabetic regimen, frequency of self-monitoring, and recent glucose or HbA1c values is essential for safe dental management. Appointments should preferably be scheduled early in the day, ensuring that patients have taken their usual medications and meals to minimize the risk of hypoglycemia. Clinicians should also be aware of the typical warning signs of hypoglycemia—such as sweating, confusion, tremors, or behavioral changes—and be prepared to manage acute episodes promptly. In conscious patients, oral glucose (via tablets, sugar solutions, or gels) should be administered, whereas unconscious patients require intramuscular glucagon and urgent medical support. Maintaining awareness of these considerations can significantly enhance the safety and quality of dental care for patients with diabetes [7].

Given the high prevalence of undiagnosed diabetes in Europe (≈22 million adults; IDF, 2021) and in Romania specifically (PREDATORR study), dental professionals may play a crucial role in early case detection. International guidelines now recommend that oral healthcare providers screen patients with periodontitis or multiple abscesses for diabetes risk and refer them to primary care for further evaluation [12,29].

Despite long-standing evidence linking diabetes and periodontal disease, awareness of this relationship remains limited among medical professionals and patients with diabetes. Previous research has shown that, while dental practitioners are generally familiar with the bidirectional association, many physicians and patients are unaware of the impact that periodontal inflammation can have on metabolic control or diabetes complications [30]. Furthermore, structural barriers between medical and dental care systems often hinder coordinated management. Nevertheless, patients with diabetes have expressed a clear desire for consistent messages from all healthcare professionals regarding the importance of oral health and for improved access to dental care [30]. In response to this knowledge gap, several international organizations have promoted interdisciplinary collaboration between dental and medical teams. The European Federation of Periodontology (EFP) issued its 2012 Manifesto on Periodontitis and General Health, urging all healthcare providers to engage in the prevention, early diagnosis, and management of periodontal disease to mitigate its impact on both oral and systemic health [31]. Subsequently, the EFP–AAP Joint Workshop on Periodontology (2013) and the EFP–IDF Workshop on Diabetes and Periodontitis (2017) produced joint consensus reports published simultaneously in dental and medical journals to increase mutual awareness among professionals [12]. These initiatives have since evolved into comprehensive guidance documents for oral healthcare providers, medical professionals, pharmacists, and policymakers, emphasizing the need for integrated care models and consistent patient education [32].

Recent findings have demonstrated that sex-related differences may influence both the prevalence and severity of periodontitis among individuals with diabetes. As highlighted in a recent study [33] male patients tend to present with deeper periodontal pockets, higher clinical attachment loss, and more severe bleeding on probing compared to females, particularly among those with poorly controlled diabetes. These differences have been attributed to behavioral and hormonal factors, as men generally exhibit poorer oral hygiene habits and higher smoking rates, whereas estrogen in females may exert a partial protective effect through modulation of vascular and inflammatory responses. These data suggest that biological sex may act as an additional modifier in the bidirectional diabetes–periodontitis relationship, potentially influencing disease expression and treatment outcomes. Incorporating sex-specific considerations into preventive and therapeutic strategies could therefore enhance the personalization of care for diabetic individuals with periodontal disease.

Age also showed a significant positive correlation with periodontal destruction, in agreement with evidence suggesting that the prevalence and severity of periodontitis increase with age due to cumulative exposure to risk factors and decreased regenerative capacity. The growing aging population, combined with increased tooth retention, may contribute to a rising burden of periodontal disease worldwide [34].

This study has several limitations that should be acknowledged. First, patients were recruited from two different sources: individuals with diabetes were referred from a hospital-based diabetes care facility, while non-diabetic controls were selected from attendees of a dental clinic. This difference in recruitment settings may introduce selection bias, as the two groups could differ in healthcare-seeking behavior or systemic health status beyond diabetes itself. Although efforts were made to achieve comparable distributions of sex and smoking status, diabetic participants were older than controls; this difference is reported transparently and should be considered when interpreting the results. In addition, both the higher mean age and greater smoking intensity observed among diabetic individuals may have acted as confounding factors, partly contributing to the differences in periodontal outcomes. Furthermore, the higher prevalence of systemic comorbidities such as hypertension and cardiovascular disease among diabetic participants may have further contributed to periodontal breakdown through shared inflammatory and vascular mechanisms, representing an additional potential confounding factor. Because the sample size was not determined by power analysis, the study may be underpowered to detect smaller effect sizes, and the findings should be interpreted accordingly. While examiners were trained under a standardized protocol and supervised by a reference examiner, formal reliability coefficients (ICC) were not recorded for all examiners/patients across the entire study period. This may introduce residual measurement variability for PPD/CAL despite the calibration and quality-control procedures. Second, the sample size, although adequate for the statistical analyses performed, remains modest relative to the national diabetic population and may not capture the full diversity of Romanian patients.

Third, smoking status and lifestyle habits were self-reported, which may be subject to recall or reporting bias. Fourth, inflammatory biomarkers (e.g., CRP, salivary cytokines) were not consistently available for all participants, limiting the exploration of biological mechanisms linking diabetes and periodontal inflammation. Given the descriptive cross-sectional design, the analyses do not support causal interpretations between glycemic control and periodontal outcomes. Multivariable regression was not performed, and although differences in age and smoking status were reported transparently, residual confounding may still influence the associations observed. In particular, the substantially higher daily cigarette consumption among diabetic participants may have confounded the observed associations between diabetes and periodontal parameters. Additionally, the inverse correlation observed between HbA1c and plaque index may represent a behavioral artifact influenced by differential healthcare supervision or oral hygiene reinforcement among patients with poorer glycemic control, rather than a true biological effect. The large standard deviations observed for CAL indicate heterogeneous disease severity within each group, which may have introduced dispersion effects on mean estimates. Future studies using site-level or median-based analyses could provide a more robust assessment of central tendency and reduce the influence of extreme values.

Finally, this study was conducted in western Romania and included patients mainly from an urban university hospital setting. Therefore, rural or underserved populations may be underrepresented, which could limit the generalizability of our findings. Future research should include longitudinal and multicentric cohorts to provide broader, population-level evidence. Given the cross-sectional design, the study does not allow inference of temporality or causation. The observed associations should therefore be interpreted as correlational rather than causal.

Despite these limitations, the study provides valuable evidence supporting the integration of periodontal and systemic health care. Promoting primary prevention and improving awareness of the oral–systemic connection may yield benefits extending beyond dental well-being, contributing to better metabolic control and potentially lowering the risk of diabetes-related complications. Our results thus support current recommendations that periodontal evaluation and therapy should be integrated into diabetes care pathways, both to improve oral health and to contribute to better systemic metabolic control. Future longitudinal and multicentric studies are warranted to further elucidate the temporal relationship between glycemic status and periodontal breakdown, assess treatment outcomes over time, and evaluate the effectiveness of community-based screening strategies. Implementing coordinated national programs that include multidisciplinary collaboration between diabetologists, primary care physicians, and dental professionals may significantly enhance early detection, improve patient adherence, and reduce the cumulative burden of chronic disease at the population level.

From a clinical perspective, these findings highlight the importance of interdisciplinary communication and awareness between dental and medical professionals in the management of patients with diabetes. Dental practitioners should routinely record a complete medical history and consider glycemic status when planning periodontal care, ensuring appropriate coordination with diabetologists or primary care physicians. Implementing shared preventive and therapeutic strategies across medical and dental care settings is therefore essential to address this bidirectional relationship effectively.

## 5. Conclusions

The present study supports the association between diabetes mellitus and significantly poorer periodontal outcomes, characterized by increased probing depths, attachment loss, and advanced disease staging. These findings suggest the critical role of glycemic control in periodontal health and indicate that periodontitis in diabetic individuals is associated with a complex interplay of systemic inflammation, local biofilm accumulation, and behavioral risk factors such as smoking. From a preventive standpoint, regular periodontal assessment and timely intervention could be beneficial components of comprehensive diabetes care. Multidisciplinary collaboration between dental and medical professionals is essential to identify at-risk patients, reduce systemic inflammatory burden, and prevent disease progression. Given the rising prevalence of both diabetes and periodontitis in Romania, further longitudinal and multicentric studies are warranted to clarify causal pathways and to inform future population-level preventive strategies.

## Figures and Tables

**Table 1:** Baseline demographic and clinical characteristics of the study population.

Variable		Diabetes Group (n = 152)	Non-Diabetic Group (n = 193)
Age, years (mean ± SD)		58.3 ± 9.7	49.6 ± 10.2
Gender, n (%)	Female	74 (48.7%)	98 (50.8%)
	Male	78 (51.3%)	95 (49.2%)
Place of residence	Urban	94 (61.8%)	134 (69.4%)
	Rural	58 (38.2%)	59 (30.6%)
Smoking status, n (%)	Never	72 (47.4%)	119 (61.7%)
	Former	25 (16.4%)	29 (15.0%)
	Current	55 (36.2%)	45 (23.3%)
Smoking intensity (cigarettes/day, median [IQR])		15 (10–20)	5 (0–10)
Systemic comorbidities, n (%)	Hypertension	102 (67.1%)	38 (19.7%)
	Cardiovascular disease (non-HTN)	28 (18.4%)	11 (5.7%)
	Respiratory disease	9 (5.9%)	4 (2.1%)
	Other systemic disease	21 (13.8%)	10 (5.2%)
	None	8 (5.3%)	145 (75.1%)
HbA1c, % (mean ± SD)		7.68 ± 1.52	5.32 ± 0.47

**Table 2:** Comparison of periodontal parameters between diabetic and non-diabetic participants.

Parameter	Diabetic Group (Mean ± SD)	Non-Diabetic Group (Mean ± SD)	Mean Difference (95% CI)	*p*-Value
Probing Pocket Depth (PPD, mm)	4.33 ± 1.22	3.70 ± 0.86	0.63 (0.45–0.81)	<0.001
Clinical Attachment Level (CAL, mm)	1.89 ± 4.70	1.70 ± 4.29	0.19 (0.01–0.37)	0.043
Plaque Index (%)	44.01 ± 29.46	25.04 ± 26.43	18.97 (12.44–25.50)	<0.001
Bleeding on Probing (%)	43.84 ± 28.76	26.61 ± 27.44	17.23 (10.86–23.60)	<0.001

**Table 3:** Distribution of periodontal stage (I–IV) and grade (A–C) by diabetes status.

Periodontal Parameter	Category	Non-Diabetic n (%)	Diabetic n (%)	*p*-Value
Stage				0.001
	I	0 (0.0%)	2 (1.3%)	
	II	14 (7.3%)	0 (0.0%)	
	III	86 (44.6%)	60 (39.0%)	
	IV	93 (48.1%)	90 (59.7%)	
Grade				0.004
	A	4 (2.1%)	0 (0.0%)	
	B	66 (34.2)	34 (21.3%)	
	C	123 (63.7%)	118 (78.7%)	

Notes: Data are presented as n (%). *p*-values are derived from chi-squared (χ^2^) tests comparing the distribution of periodontal stages (I–IV) and grades (A–C) between diabetic and non-diabetic periodontal patients. Classification according to the 2018 EFP/AAP criteria.

**Table 4:** Medication profile among study participants.

Medication Category	Diabetic Group (n = 152)	Non-Diabetic Group (n = 193)
Metformin-based oral agents	108 (71.1%)	-
Insulin therapy	42 (27.6%)	-
Combination (insulin + oral)	31 (20.4%)	-
Antihypertensive agents	97 (63.8%)	29 (15.0%)
Lipid-lowering drugs (statins)	84 (55.3%)	-
Antiplatelet/anticoagulant therapy	19 (12.5%)	8 (4.1%)
Respiratory medications	-	5 (2.6%)
No systemic medication	-	138 (71.5%)

Notes: Data are presented as n (%). Percentages are based on the number of participants in each group. Medication use was self-reported and verified through available medical records.

**Table 5:** Distribution of local periodontal risk factors.

Local Risk Factor	Diabetic Group (n = 152)	Non-Diabetic Group (n = 193)
Dental plaque	142 (93.4%)	158 (81.9%)
Calculus deposits	128 (84.2%)	131 (67.9%)
Faulty restorations	97 (63.8%)	88 (45.6%)
Cervical/root caries	91 (59.9%)	76 (39.4%)
Prosthetic maladaptation	62 (40.8%)	58 (30.1%)
Malpositioned teeth	49 (32.2%)	55 (28.5%)

Notes: Data are presented as n (%). Each local periodontal risk factor was recorded as present or absent per participant based on clinical examination. Percentages are calculated relative to the total number of individuals in each group.

## Data Availability

The data presented in this study are available on request from the corresponding author.

## Abbreviations

The following abbreviations are used in this manuscript:

DM	Diabetes Mellitus
T1DM	Type 1 Diabetes Mellitus
T2DM	Type 2 Diabetes Mellitus
HbA1c	Glycated Hemoglobin
PPD	Probing Pocket Depth
CAL	Clinical Attachment Level
PI	Plaque Index
BOP	Bleeding on Probing
CRP	C-Reactive Protein
NCD	Non-Communicable Disease
ROS	Reactive Oxygen Species
AGE	Advanced Glycation End-product
TLR	Toll-like Receptor
SPSS	Statistical Package for the Social Sciences
EFP	European Federation of Periodontology
IDF	International Diabetes Federation
CPITN	Community Periodontal Index of Treatment Needs
IQR	Interquartile Range
SD	Standard Deviation

## References

[1] Lim G., Janu U., Chiou L.-L., Gandhi K.K., Palomo L., John V. (2020). Periodontal Health and Systemic Conditions. Dent. J..

[2] Eke P.I., Thornton-Evans G.O., Wei L., Borgnakke W.S., Dye B.A., Genco R.J. (2018). Periodontitis in US Adults. J. Am. Dent. Assoc..

[3] El Chaar E. (2025). Periodontal Disease: A Contributing Factor to Adverse Outcome in Diabetes. J. Diabetes.

[4] Costa K.L., Taboza Z.A., Angelino G.B., Silveira V.R., Montenegro R., Haas A.N., Rego R.O. (2017). Influence of Periodontal Disease on Changes of Glycated Hemoglobin Levels in Patients with Type 2 Diabetes Mellitus: A Retrospective Cohort Study. J. Periodontol..

[5] Huang Y., Guo W., Zeng J., Chen G., Sun W., Zhang X., Tian W. (2016). Prediabetes Enhances Periodontal Inflammation Consistent with Activation of Toll-Like Receptor–Mediated Nuclear Factor-κB Pathway in Rats. J. Periodontol..

[6] Alasqah M., Mokeem S., Alrahlah A., Al-Hamoudi N., Abduljabbar T., Akram Z., Vohra F., Javed F. (2018). Periodontal Parameters in Prediabetes, Type 2 Diabetes Mellitus, and Non-Diabetic Patients. Braz. Oral Res..

[7] Preshaw P.M., Bissett S.M. (2019). Periodontitis and Diabetes. Br. Dent. J..

[8] El-Sharkawy H.M., Anees M.M., Van Dyke T.E. (2016). Propolis Improves Periodontal Status and Glycemic Control in Patients with Type 2 Diabetes Mellitus and Chronic Periodontitis: A Randomized Clinical Trial. J. Periodontol..

[9] Taşdemir Z., Özsarı Taşdemir F., Koçyiğit İ., Yazıcı C., Gürgan C.A. (2016). The Clinical and Systemic Effects of Periodontal Treatment in Diabetic and Non-Diabetic Obese Patients. J. Oral Sci..

[10] Graziani F., Gennai S., Solini A., Petrini M. (2018). A Systematic Review and Meta-analysis of Epidemiologic Observational Evidence on the Effect of Periodontitis on Diabetes An Update of the EFP—AAP Review. J. Clin. Periodontol..

[11] Madianos P.N., Koromantzos P.A. (2018). An Update of the Evidence on the Potential Impact of Periodontal Therapy on Diabetes Outcomes. J. Clin. Periodontol..

[12] Sanz M., Ceriello A., Buysschaert M., Chapple I., Demmer R.T., Graziani F., Herrera D., Jepsen S., Lione L., Madianos P. (2018). Scientific Evidence on the Links between Periodontal Diseases and Diabetes: Consensus Report and Guidelines of the Joint Workshop on Periodontal Diseases and Diabetes by the International Diabetes Federation and the European Federation of Periodontology. J. Clin. Periodontol..

[13] Polak D., Shapira L. (2018). An Update on the Evidence for Pathogenic Mechanisms That May Link Periodontitis and Diabetes. J. Clin. Periodontol..

[14] Herrera D., Sanz M., Shapira L., Brotons C., Chapple I., Frese T., Graziani F., Hobbs F.D.R., Huck O., Hummers E. (2023). Association between Periodontal Diseases and Cardiovascular Diseases, Diabetes and Respiratory Diseases: Consensus Report of the Joint Workshop by the European Federation of Periodontology (EFP) and the European Arm of the World Organization of Family Doctors (WONCA Europe). J. Clin. Periodontol..

[15] D’Aiuto F., Gkranias N., Bhowruth D., Khan T., Orlandi M., Suvan J., Masi S., Tsakos G., Hurel S., Hingorani A.D. (2018). Systemic Effects of Periodontitis Treatment in Patients with Type 2 Diabetes: A 12 Month, Single-Centre, Investigator-Masked, Randomised Trial. Lancet Diabetes Endocrinol..

[16] Simpson T., Needleman I., Wild S.H., Moles D.R., Mills E.J., The Cochrane Collaboration (2004). Treatment of Periodontal Disease for Glycaemic Control in People with Diabetes. Cochrane Database of Systematic Reviews.

[17] Borgnakke W.S., Poudel P. (2021). Diabetes and Oral Health: Summary of Current Scientific Evidence for Why Transdisciplinary Collaboration Is Needed. Front. Dent. Med..

[18] Mota M., Popa S.G., Mota E., Mitrea A., Catrinoiu D., Cheta D.M., Guja C., Hancu N., Ionescu-Tirgoviste C., Lichiardopol R. (2016). Prevalence of Diabetes Mellitus and Prediabetes in the Adult R Omanian Population: PREDATORR Study. J. Diabetes.

[19] Natarajan P., Madanian S., Marshall S. (2025). Investigating the Link between Oral Health Conditions and Systemic Diseases: A Cross-Sectional Analysis. Sci. Rep..

[20] George A., Poudel P., Kong A., Villarosa A., Calache H., Arora A., Griffiths R., Wong V.W., Gussy M., Martin R.E. (2022). Developing and Pilot Testing an Oral Health Screening Tool for Diabetes Care Providers. BMC Prim. Care.

[21] Dumitrescu R., Bolchis V., Popescu S., Ivanescu A., Bolos A., Jumanca D., Galuscan A. (2025). Oral Health and Quality of Life in Type 2 Diabetic Patients: Key Findings from a Romanian Study. J. Clin. Med..

[22] Bolchis V., Jumanca D., Dumitrescu R., Balean O., Toderas N.A., Popescu S., Marcu A., Marian C., Galuscan A. (2025). Glycemic Control, Inflammatory Mediators, and Periodontal Health: A Cross-Sectional Study in Patients with Diabetes. J. Clin. Med..

[23] Bolchis V., Alexa I., Toderas N.A., Dumitrescu R., Sava-Rosianu R., Balean O., Alexa V.T., Popescu S., Jumanca D., Galuscan A. (2025). Associations Between Lifestyle Factors, Oral Health Behaviors, and Glycemic Control in Type 2 Diabetic Patients. J. Clin. Med..

[24] Caton J.G., Armitage G., Berglundh T., Chapple I.L.C., Jepsen S., Kornman K.S., Mealey B.L., Papapanou P.N., Sanz M., Tonetti M.S. (2018). A New Classification Scheme for Periodontal and Peri-implant Diseases and Conditions—Introduction and Key Changes from the 1999 Classification. J. Clin. Periodontol..

[25] Genco R.J., Sanz M. (2020). Clinical and Public Health Implications of Periodontal and Systemic Diseases: An Overview. Periodontol. 2000.

[26] Libby P. (2021). The Changing Landscape of Atherosclerosis. Nature.

[27] Preshaw P.M., Alba A.L., Herrera D., Jepsen S., Konstantinidis A., Makrilakis K., Taylor R. (2012). Periodontitis and Diabetes: A Two-Way Relationship. Diabetologia.

[28] Zhuang J., Ren Y., Chen M., Yue M., Yuan C., Duan R. (2025). Efficacy of Localized Sustained-Release Drugs in Periodontitis and Comorbid Diabetes: A Systematic Review and Meta-Analysis. iScience.

[29] Orlandi M., Muñoz Aguilera E., Marletta D., Petrie A., Suvan J., D’Aiuto F. (2022). Impact of the Treatment of Periodontitis on Systemic Health and Quality of Life: A Systematic Review. J. Clin. Periodontol..

[30] Kudoh R., Shibayama T. (2025). Attitudes and Practices of Dental Hygienists Regarding Diabetes Screening and Medical–Dental Collaboration: A Nationwide Cross-Sectional Study in Japan. Healthcare.

[31] European Federation of Periodontology The EFP Manifesto: Perio and General Health. https://www.efp.org/about-the-efp/who-we-are-what-we-do/efp-manifesto/.

[32] Herrera D., Berglundh T., Schwarz F., Chapple I., Jepsen S., Sculean A., Kebschull M., Papapanou P.N., Tonetti M.S., Sanz M. (2023). Prevention and Treatment of Peri-implant Diseases—The EFP S3 Level Clinical Practice Guideline. J. Clin. Periodontol..

[33] Schulze A., Busse M. (2016). Gender Differences in Periodontal Status and Oral Hygiene of Non-Diabetic and Type 2 Diabetic Patients. Open Dent. J..

[34] Graziani F., Tsakos G. (2020). Patient-based Outcomes and Quality of Life. Periodontol. 2000.

